# Literary Notes

**Published:** 1900-04

**Authors:** 


### LITERARY NOTES.

THE *Anglo-Saxon Review*, Lady Randolph Churchill's superb quarterly, in its current issue contains an article on Chinese Doctors and Medical Treatment, by Isabella L. Bishop, which abounds in many interesting details. She says among other things, that "a fashionable doctor is carried in a sedan chair by three or four bearers, who simulate great haste. He wears a most oracular expression, and is apparently always absorbed in consulting books and notes. He is received with much ceremony. If the patient be a lady a bamboo screen conceals her from him. After a prolonged examination of the 'twelve pulses' has acquainted him with all he desires to know he writes a prescription."

Some physicians in this country are pretty good imitators of this kind of a doctor, except, perhaps, the sedan chair. The entire article is interesting.

THE *Columbus Medical Journal*, heretofore for several years having been published twice a month, has returned to the monthly issue plan. We have no doubt the change is a wise one and hope our excellent Ohio contemporary will continue to reap the full measure of reward that it deserves, as a high-class medical periodical.

THE *Bookman* is publishing as a serial story for 1900, a novel by an American author, Mr. John Uri Lloyd, who, although unknown as yet as a writer of fiction, is believed to deserve a foremost place among the newer American novelists. The story is entitled, Stringtown on the Pike, and began in the March number of *The Bookman*, to continue through about ten issues of the magazine.

The first eight chapters indicate the general trend of the story and show its distinctively American type. The plot is laid in a Kentucky hamlet and its characters are such as might live in such a place—real live Americans. There isnothing impossible in the tale; its heroine, the Judge, the Professor, the Colonel, the Clergyman, the old villagers, and Cupe, are all probabilities in such a village and a part of its every-day life.

Mr. Lloyd made a strong impression upon the literary world with "Etidorhpa" a few years ago, and his fame is likely to be enhanced by Stringtown on the Pike. A subscription to *The Bookman* is an excellent way to secure its early reading, besides much other material published in every number of great interest.

THE Mütter lectureship of the College of Physicians of Philadelphia for 1902-3 is under preparation, according to an announcement lately issued by the chairman, Dr. John H. Brinton.

This is a course of ten lectures instituted by the late Professor Thomas Dent Mütter, M.D., LL.D., on some Point or points in surgical pathology, to be delivered in the winter of 1902-03 before the College of Physicians of Philadelphia.

The compensation is $600. The appointment is open to the profession at large. Applications, stating in full subjects of proposed lectures, must be made before October 1, 1900, to the Committee on the Mütter Museum, corner 13th and Locust Street, Philadelphia.

---

THE Samuel D. Gross prize of $1,000 is again offered for competition. No essay which the trustees deemed worthy of the prize having been received on January 1, 1900, they hereby announce that the prize will be awarded on October 1, 1901.

The conditions annexed by the testator are that the prize "shall be awarded every five years to the writer of the best original essay, not exceeding 150 printed pages, octavo in length, illustrative of some subject in surgical pathology or surgical practice, founded upon original investigations, the candidates for the prize to be American citizens."

It is expressly stipulated that the competitor who receives the prize, shall publish his essay in book form, and that he shall deposit one copy of the work in the Samuel D. Gross Library of the Philadelphia Academy of Surgery, and that on the title page, it shall be stated that to the essay was awarded the Samuel D. Gross prize of the Philadelphia Academy of Surgery.

The essays which must be written by a single author in the English language, should be sent to the "Trustees of the Samuel D. Gross Prize of the Philadelphia Academy of Surgery, care of the College of Physicians, 219 S. 13th St., Philadelphia," on or before October 1, 1901.

Each essay must be distinguished by a motto, and accompanied by a sealed envelope bearing the same motto, and containing the name and address of the writer. No envelope will be opened except that which accompanies the successful essay. The committee will return the unsuccessful essays if reclaimed by their respective writers, or their agents, within one year. The committee reserves the right to make no award if the essays submitted are not considered worthy of the prize.

Drs. W. W. Keen, J. Ewing Mears and J. Chalmers Da Costa, are the trustees.

---

THE *Bibliographia Medica* is the name given a new publication, the first number of which was issued February 15, 1900. It is constructed on the plan of the *Index Medicus* which was given up for non-support. The new index will appear monthly, issued from the Institut de Bibliographie, 93 Boulevard, St. Germain, Paris. The subscription price to foreigners is sixty francs. The editor-in-chief is Dr. Marcel Baudouin. This commendable enterprise deserves the support of all progressive men in the profession of medicine.

---

THE Board of Trustees of the University of Pennsylvania announces that it has finally secured entire control of the *University Medical Magazine*, and that in the future it will be published under the auspices and with the support of the University. Heretofore the Magazine has been in the hands of a stock company, over which the University had no control.

Owing to the necessary delay attending the transfer of the property, and also owing to the time required for a thorough reorganisation, it will be impossible to issue numbers for January or February; the first number of the year appeared in March.

It is proposed that the magazine shall hereafter be the organ of the Department of Medicine, and that it shall be creditable to that department. In every way its readers will be kept fully informed as to the work that is being done in the clinic, the laboratory and the hospital, by the members of the teaching and hospital staff. Contributions upon subjects allied to medicine will be secured from those engaged in such work at the University.

The liberal policy adopted by the management will enable the magazine in the future to obtain at all times the most desirable material for publication. Dr. Charles H. Frazier has recently been appointed editor, and will at once assume the duties of his office.

